# Comparison of the skeletal, dentoalveolar, and periodontal changes after Ni–Ti leaf spring expander and rapid maxillary expansion: a three-dimensional CBCT based evaluation

**DOI:** 10.1007/s00784-023-05144-6

**Published:** 2023-07-19

**Authors:** Andrea Abate, Alessandro Ugolini, Cinzia Maspero, Francesca Silvestrini-Biavati, Alberto Caprioglio, Valentina Lanteri

**Affiliations:** 1 Department of Biomedical Surgical and Dental Sciences, University of Milan, 20142 Milan, Italy; 2 Fondazione IRCCS Cà Granda, Ospedale Maggiore Policlinico, 20142 Milan, Italy; 3 Department of Sciences Integrated Surgical and Diagnostic, University of Genova, Genoa, Italy

**Keywords:** Malocclusion, Cone beam computed tomography, Rapid maxillary expansion, Slow maxillary expansion, Leaf Expander

## Abstract

**Background:**

The aim of the present study was twofold:(1) three-dimensionally evaluate the quantitative skeletal and dentoalveolar changes after Ni–Ti leaf spring expander (leaf expander) and rapid maxillary expansion (RME) in mixed dentition patients;(2) analyze the modifications of the buccal alveolar bone plate of the maxillary first permanent molars.

**Methods:**

Patients who underwent CBCT scans before and after maxillary expansion were randomly selected from the records archived at the Department of Biomedical Surgical and Dental Sciences, University of Milan, Italy.

Inclusion criteria were the following: no systemic disease or syndromes; maxillary transverse deficiencies (difference between the upper intermolar width and the lower intermolar width of at least 3 mm and/or clinical need based on radiographic evaluation), early mixed dentition with ages between 7 to 10 years old; cervical vertebra maturation stage (CVMS) 1 or 2; no pathologic periodontal status; skeletal class I or II; maxillary expander cemented on the upper second deciduous molars. Exclusion criteria were the following: patients with pubertal or post-pubertal stage of development (CVMS 3–6); late deciduous or late mixed dentition, impossibility to use the second primary molar as anchorage; skeletal class III malocclusion; craniofacial syndromes; patients unable to be followed during the treatment period. Twenty-three patients treated with Leaf Expander, 11 males (mean age 7.8 ± 0.6 years) and 12 females (mean age 8.1 ± 0.8 years), met the inclusion criteria and constituted the case group. Twenty-four (control group) treated with conventional RME, 12 males (mean age 8.4 ± 0.9 years) and 12 females (mean age 8.1 ± 0.7 years). The paired-sample T test was used for intra-group comparison to evaluate the difference between before (T1) and after (T2) maxillary expansion. Independent sample t-test was computed to perform between groups comparison of the skeletal, dentoalveolar, and periodontal changes.

**Results:**

The Leaf Expander and RME group showed a significant increase between T1 and T2 for most of the skeletal and dentoalveolar variables. Concerning the skeletal variables only the RME demonstrated a significant increase at the level of the posterior nasal (PNW) and apical base width (PABW) and maxillary mid-alveolar width (MMW). Despite this, when compare with the Leaf Expander, the RME group exhibited a statistically larger width increase for only two skeletal parameters: PNW (*p* = 0.03) and MMW (*p* = 0.02). No significant changes at the periodontal level were found in either group.

**Conclusions:**

According to the current research, the authors confirm the effectiveness of the Leaf Expander and RME to produce similar skeletal and dentoalveolar effects in mixed dentition subjects. Moreover, the devices anchored to deciduous teeth did not reduce the thickness and height of the buccal bone at the level of the maxillary permanent first molars in either of the two groups.

## Background

Maxillary transverse deficiency is one of the most significant examples of malocclusion occurring during deciduous and mixed dentition and is often accompanied by unilateral or bilateral posterior crossbite [1]. Some previous studies have suggested that it occurs in 8–20% of children [2, 3].

The transverse maxillary contraction caused by dental, skeletal, or neuromuscular components are regarded as the main etiological factors for posterior crossbite. This malocclusion may cause a functional mandibular shift and, if left untreated, could produce mandibular skeletal asymmetries and long-term effects on the development of craniofacial structures and their functions [4].

Many studies underline the importance of early diagnosis and early treatment [5, 6] by means of palatal expansion treatment [7]. This has been largely adopted to resolve posterior crossbite and maxillary hypoplasia by opening the mid-palatal suture and producing a proper increase of the maxillary width, thus helping to decrease the severity of future crowding in growing subjects [8, 9].

Over the years, various types of protocols have been tested in an effort to obtain the best results for mixed dentition. Examples of this include tooth-borne devices with different expansion activations like rapid maxillary expansion (RME), slow maxillary expansion (SME), and semi-rapid maxillary expansion. Appliances with a rigid screw can be activated following rapid/slow or semi-rapid maxillary expansion protocols depending on the frequency of screw activation.

Several studies have investigated the effects of RME and SME and both of the approaches seem to determine a transverse change in the maxilla, according to the literature [8, 10, 11]. Despite this, no significant evidence is present in the literature as to which appliance or screw activation protocols are the best to achieve the maximum skeletal expansion with the least side effects[12–14]. RME produce an immediate mid-palatal suture separation using heavy and intermittent forces for a short time which produce a significant effect on maxillary transverse dimensions [15], whilst, in contrast, SME is done using intermittent and lower forces for a longer period of time [10]. On the contrary, appliances with a Ni–Ti elastic modulus produce a slow maxillary expansion using low and constant forces and are more comfortable for young patients and do not require parental collaboration [16].

Recently, a new slow palatal expander with Ni–Ti leaf springs (Leaf Expander®, Leone, Italia) as an active part has been introduced [17]. The design of the device is similar to a Hyrax expander, the difference being that the Leaf Expander has nickel titanium leaf springs through which lower, more steady and calibrated forces are produced to obtain the palatal expansion [16]. The main objective of the appliance is to obtain a compliance-independent SME with an appropriate force system [18]. It eliminates the need for home activation with no compliance from patients’ parents and simplifies clinical management. It performs controlled tooth movement for expansion and avoids undesirable side effects on the permanent teeth [19]. Literature reports that the Leaf Expander, compared to the conventional RME, is less painful and able to produce an analogous amount of expansions [20, 21].

Among the different radiographic analyses, cone-beam computed tomography (CBCT) has acquired growing popularity in the last decade due to its several advantage points, while also maintaining relatively low doses of ionizing radiation [22, 23].

Published data seems hopeful [24–27] but no research has been conducted on CBCT to evaluate the orthopedic, dentoalveolar, and periodontal changes.

The aim of the present CBCT based-retrospective study was twofold: firstly to three-dimensionally evaluate the quantitative skeletal and dentoalveolar changes after Ni–Ti leaf spring expansion (Leaf Expander) and rapid maxillary expansion (RME) in mixed dentition patients; and secondly, analyze the modifications of the buccal alveolar bone plate of the maxillary first permanent molars induced by the two different maxillary expansion protocols. The null hypothesis was that there are no differences in the skeletal, dentoalveolar, and periodontal effects between the two groups.

## Methods

This is a retrospective study on skeletal, dentoalveolar, and periodontal changes after Leaf Expander and conventional RME palatal expansion treatment, analyzing the CBCTs of subjects who underwent orthodontic treatment at Department of Biomedical Surgical and Dental Sciences, University of Milan, Italy, from March 2018 to June 2020. The protocol of the current research was approved by the Ethical Committee of the Fondazione IRCCS Ca’Granda, Ospedale Maggiore, Milan - Italy (protocol n.573/15) and all the procedures performed in the present study were in accordance with the 1964 Helsinki declaration and its later amendments or comparable ethical standards. As a routine procedure, a signed informed consent for releasing diagnostic records in anonymous form for scientific purposes was obtained from the parents of all of the patients prior to the start of the treatment.

### Participants and eligibly criteria

CBCT scans of patients taken before and after maxillary expansion for different medical reasons were randomly chosen from the record archives of the Department of Biomedical Surgical and Dental Sciences, University of Milan, Italy. To be included in the study, patients had to comply with the following inclusion criteria: no systemic disease; maxillary transverse deficiencies(based on the difference between the upper intermolar width and the lower intermolar width of at least 3 mm or in case this difference was lower, based on clinical need after radiographic evaluation); early mixed dentition of those with ages from 7 to 10 years old; cervical vertebra maturation stage CVMS 1 or 2 according to McNamara classification [28]; first upper permanent molars fully erupted; no pathologic periodontal status; skeletal class I or II according to Steiner’ classification[29]; maxillary expander (RME or Leaf Expander) cemented on the upper second deciduous molars[30]. Exclusion criteria were the following: patients with age older than 12 years with a pubertal or post-pubertal stage of development (CVMS 3–6); late deciduous or late mixed dentition, impossibility to use the second primary molar as anchorage (agenesis of upper second premolars or important carious lesions); subjects presenting skeletal class III malocclusion; cleft lip and/or palate and craniofacial syndromes; patients with any other orthodontic treatment during the period of maxillary expansion and the retention period.

Moreover, CBCT of subjects without sufficient clarity in identifying landmarks were excluded.

The prerequisite for carrying out this research was that the first CBCT scan (T1) had to have been done at max one month before the maxillary expansion, and that the second one (T2) had to have been taken out about 8.5 months (varying between 7 and 9 months) after the activation phase. The medical records of 23 patients treated with the Leaf Expander met the inclusion criteria and were included into the case group which was composed of 11 males (mean age 7.8 ± 0.6 years old) and 12 females (mean age 8.1 ± 0.8 years old). The mean duration between the pre and post treatment CBCT images was 9.4 months.

The medical records of 24 patients, 12 males (mean age 8.4 ± 0.9 years old), and 12 females (mean age 8.1 ± 0.7 years old) who underwent RME therapy with the Hyrax expander and met all the inclusion criteria were included into the control group. The mean duration between the pre and post treatment CBCT images was 8.6 months.

### Maxillary expander design and activation protocols

A glass-ionomer cement (Multi-Cure; Unitek, Monrovia, CA, USA) was used to bond the devices to the deciduous second molars [31], both of which presenting wire extensions lingually to the maxillary primary canines with no posterior extension to the maxillary permanent first molars.

**Fig. 1:**
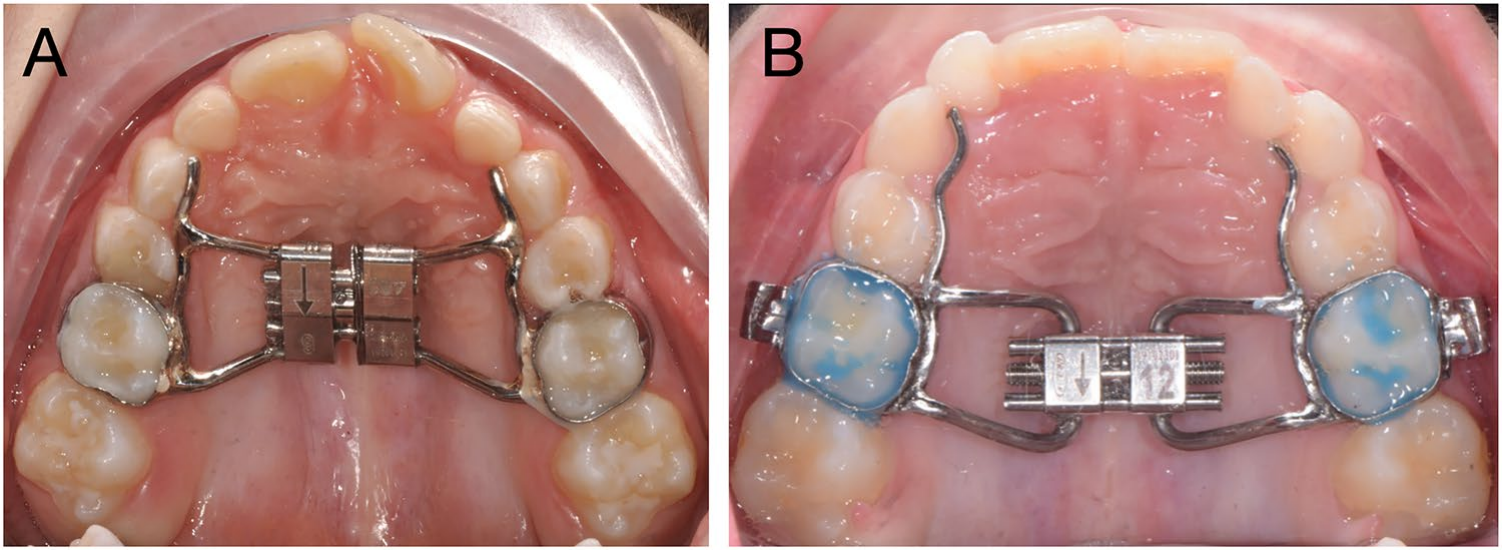
**A** Ni–ti leaf springs expander (Leaf Expander) with anterior arms extended up to the deciduous canines. **B** Conventional rapid maxillary expander (RME)

Briefly, the Leaf expander is similar to a conventional rapid palatal expander but instead of being formed medially by a jackscrew, it consists of double nickel-titanium leaf springs (Fig. 1A).

When the leaf springs is activated, it delivers a maximum expansion of 6 or 9 mm, and it generates a constant force of 450 g.

The maxillary expansion protocol for the Leaf Expander was as follows: at the moment of cementation, the device was pre-activated by the laboratory to deliver 3 or 4.5 mm of expansion, after which the re-activation (compressing the leaf springs) was performed by the clinician in the office at subsequent appointments giving the screw activation 10-quarter turns (one-quarter turn corresponds to 0.1 mm) per month until the expansion was achieved.

With regards to the RME protocols, the 10-mm screw of the palatal expander was initially turned two times (0.45-mm initial transversal activation) by the clinician immediately after cementation (Fig. 1B). Afterwards, parents of the patients were instructed to turn the screw twice directly at home per each following day (0.45-mm activation per day). After that, the clinician waited 1 week to reassess the situation and to decide whether to terminate or continue the screw activations in order to obtain a complete expansion.

In both protocols, the maxillary expansion was performed until dental overcorrection, defined as the lingual cusps of the upper first permanent molars occluding onto the buccal cusps of the lower first permanent molars, as described by Caprioglio et al. [32]. The screw, when needed, was then locked with a ligature wire and in all cases the expander was kept in situ as a passive retainer for the following eight months. In the Leaf Expander group, the retention period began 3 months after the start of the active phase (average 9 months from bonding to debonding). During this period, none of the patients underwent any further orthodontic treatment.

### Records examination and study procedure

Each patient included in the investigation was scanned using the same i-CAT CBCT Unit (Imaging Sciences International, 1910 N. Penn Road, Hatfield, PA 19440).

Isotropic voxel size of 0.4 mm, 8.9 s, and a field of view (FOV) of 9 × 11 cm, to minimize radiation exposure, 120 kV, and 20 mA were used for the acquisition protocols. The thickness of the slices was 0.4 mm which guaranteed a precise acquisition of the anatomical structures. During each registration, patients were instructed to remain in the natural head position maintaining the Frankfurt horizontal plane parallel to the floor.

The CBCT images were recorded into DICOM format (Digital Imaging and Communications in Medicine) and for each DICOM file the analysis of the effects of maxillary expansion at the skeletal, dentoalveolar level was computed using Mimics Research software version 20.0 (NV, Technologielaan 15, 3001 Leuven, Belgium, https://www.materialise.com/en/medical/mimics-innovation-suite/mimics). A second medical images viewer, OsiriX Medical Imaging 32-bit software (open source; Pixmeo, Geneva, Switzerland; www.osirix-viewer.com), was used to evaluate the buccal alveolar bone changes in both maxillary expansion protocols.

An expert orthodontist in three-dimensional imaging (AU) performed all the measurements; further, the group to which each patient had been inserted remained unknown.

The sagittal, coronal and axial planes were traced to achieve a three-dimensional orientation of the reference plane so as to ensure that the two-dimensional slices were correctly oriented as described by Miner et al. [33]: the functional occlusal plane traced as the line that combine the contact points between the upper and lower molars was used as the axial plane; the coronal plane was orthogonal to the axial one, passing through the vestibular pit of the upper first right molar; the sagittal plane was perpendicular to both the previous planes (axial and coronal), passing through the mid-point between the medial edges of the orbits. By utilizing the same coordinate system on the CBCT scans at T1 and T2 the measurement errors were likely to be greatly reduced.

### Skeletal and dentoalveolar measurements

Measurements were extrapolated from different three-dimensional transverse analyses present in the scientific literature to propose the most complete evaluation of the orthopedic and dentoalveolar effects [8, 10, 33, 34]. Thus, a proper and complete three-dimensional transverse analysis was created (Table 1).

**Table 1:** Definition of the variables used in the tomographic evaluation for the skeletal, dento-alveolar and periodontal effects

Abbreviations	Measurements	Description	
Skeletal effects			
PNW (mm)	Posterior nasal width	Maximum distance between right and left nasal cavity	
PABW (mm)	Posterior apical base width	The distance between the buccal external contours of the maxilla at the same level of the nasal cavity	
MW (mm)	Maxillary width	The distance between the right and left most superior aspects of the concavity of the maxillary bone as it joins the zygomatic process	
MMW (mm)	Maxillary mid-alveolar width	The distance measured from the right and left S points (point on the palatal cortex of the maxilla at a vertical level halfway between the buccal alveolar crest and the buccal root apex of the maxillary first molar) on the alveolar bone at the palatal mid-alveolar level	
PD (mm)	Palatal depth	The distance measured perpendicular to the upper intermolar width at its midpoint to the highest point of the palate	
Dento-alveolar effects			
PAPW (mm)	Posterior alveolar process width	Distance between the right and left alveolar crests, measured at their most inferior limits at the level of the first maxillary permanent molar	
UIMW (mm)	Upper intermolar width (16–26)	Distance between the right and left mesiopalatal cusp tips of the first permanent molars	
USIMW (mm)	Upper second intermolar width (17–27)	Distance between the right and left mesiopalatal cusp tips of the right and left second permanent molars	
LIMW (mm)	Lower intermolar width (36–46)	Distance between right and left deepest concavity between buccal and lingual cusps of first mandibular molars	
UICW (mm)	Upper intercanine width	Distance between the cusp tips of maxillary primary canines	
LICW (mm)	Lower intercanine width	Distance between the cusp tips of mandibular primary canines	
UMAA (°)	First maxillary molar axial angle (right and left)	It refers to the angle between the long axis of the maxillary first permanent molar and functional occlusal plane	
UAP (mm)	Upper arch perimeter	Measured joining contact points from right first molar to left first molar in upper arch	
LAP (mm)	Lower arch perimeter	Measured joining contact points from right first molar to left first molar in lower arch	
UCI (°)	Upper central incisor angle	It refers to the angle between the long axis of upper central incisors and bispinal plane	
Periodontal (buccal alveolar bone) effects			
BABT (mm)	Buccal alveolar bone thickness		Distance between the outer surface of the buccal alveolar plate and the outer Alveolar bone thickness wall of the buccal root at the level of the furcation
BABH (mm)	Buccal alveolar bone height		Defined as the distance between the buccal or mesio-buccal cusp tip and the buccal alveolar bone crest

**Fig. 2:**
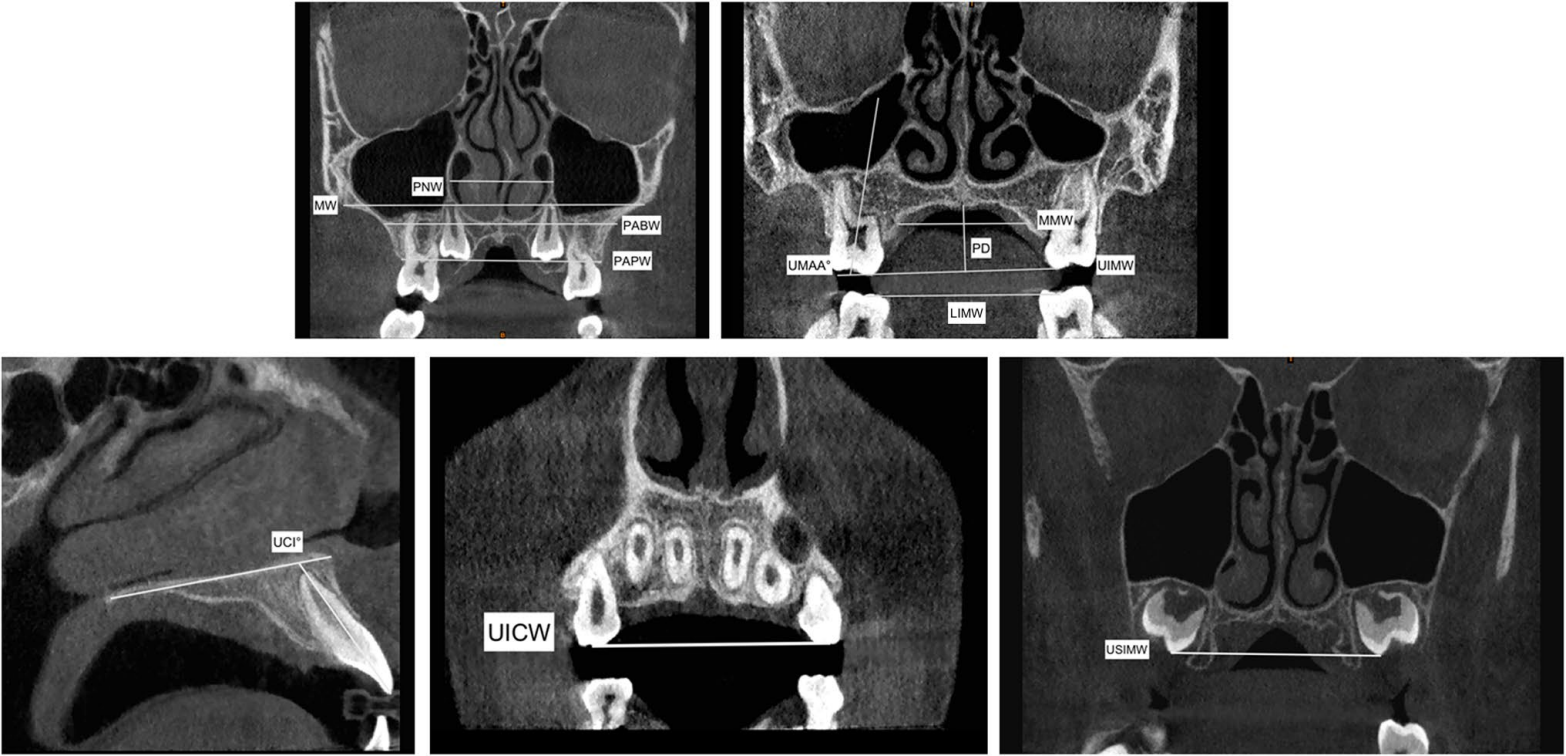
Representation of the skeletal and dentoalveolar measurements used in the present study evaluated on the CBCTs coronal and sagittal view

Dento-skeletal measurements were performed on the coronal cross-sections of the CBCT scans by passing through the center of the maxillary and mandibular primary first molar crown in accordance to Miner et al. [33] (Fig. 2). Further, an evaluation of the slices around the permanent upper first molar was often necessary to analyze the inclination of the mesio-buccal root long axis. Dental parameters relative to the width of the second permanent molars and the inclination of the central incisor were also evaluated. Moreover, the change in the palatal depth after both the expansion protocols was assessed according to Bruder et al. [34] (Fig. 2).

**Fig. 3:**
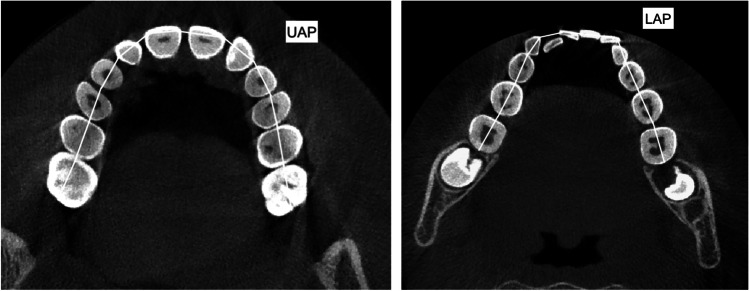
Upper arch perimeter (UAP) and lower arch perimeter (LAP) measurements traced on the CBCTs’ axial section

The CBCT axial cross section was used to measure the upper and lower arch perimeter before(T1) and after(T2) maxillary expansion (Fig. 3).

All the measurements used in the present study are summarized in Table 1.

### Periodontal measurements

Buccal alveolar bone parameters were measured before and after maxillary expansion treatment as previously described by Park et al. [35]. The images were imported into OsiriX Medical Imaging software and reoriented with the palatal plane parallel to the floor in the sagittal and coronal planes. To obtain the standardization of the slices at time T1 and T2, the scans were initially displayed in multiplanar reconstruction mode (MPRM) and then the coronal scans were set perpendicular to the mid sagittal plane by passing through the buccal/mesiobuccal cusps and the furcations of the first maxillary molars.

**Fig. 4:**
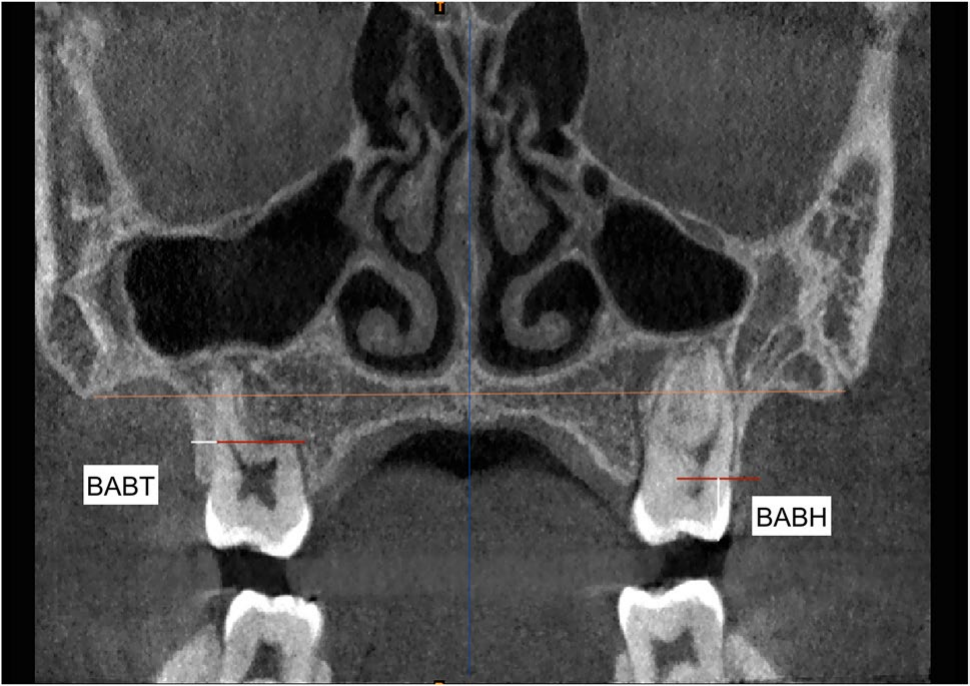
Buccal alveolar bone thickness (BABT) and buccal alveolar bone height (BABH) variables representing the characteristics of the buccal alveolar bone plate assessed on the coronal view of the CBCT scans

Changes in the buccal alveolar bone height (BABH) and thickness (BABT) were measured on the right and left side in the full-screen mode using the coronal section of each scan (Fig. 4, Table 1). As reported by Park et al. [35], BABT was determined as the distance from the vestibular surface of each root to the external surface of the buccal alveolar bone by following along a horizontal line passing through the furcation. Instead, BABH was calculated as the distance from the buccal/mesiobuccal tip to a horizontal line passing through the buccal alveolar bone ridge and perpendicular to the mid sagittal plane(Fig. 4, Table 1).

### Statistical analysis

The G*Power free software (version 3.1.9.4, Franz Faul, Universitat Kiel, Kiel, Germany) was initially used to obtain data for the power analysis calculation. The values of the mean difference in the posterior apical base width variable (PABW) before and after RME and SME obtained by Ribeiro et al. [10] were used to perform the power analysis calculation along with the corresponding SDs. To compute the analysis the following data points were used: RME group mean difference = 2.72; *σ* = 0.90, SME group mean difference = 1.79; *σ* = 0.82. The power analysis evaluation reported that to reach 80% power, 15 patients were necessary for each group. As reported above, the authors were able to select at least 23 subjects in each group increasing the strength of the present data.

IBM SPSS Statistics ver. 25.0 software (IBM Co., Armonk, NY, USA) was employed to perform the statistical analysis. Shapiro–Wilk test revealed a normal distribution of the data and, therefore, parametric tests were used for the statistical comparison. Data used in the statistical analysis were expressed as mean ± standard deviation (SD).

The independent sample *t*-test was used to compare the pre-treatment groups. The paired *t*-test was used to perform within group comparisons, assessing the difference between T1 and T2 for all the measurements in both groups.

The independent sample *t*-test was computed to perform between groups comparison of the skeletal, dentoalveolar, and periodontal changes that occurred after RME and Leaf protocols for all the variables considered.

To assess the intra-examiner and inter-examiner reliability of measurements, intraclass correlation coefficient (ICC) was computed. CBCT scans of 15 randomly selected patients were reoriented and measured a second time by a second investigator (A.A) after a minimum of 30 days. The entire process was then repeated by the first researcher (AU) with no knowledge of the first measurements. Moreover, Dahlberg’s formula [36] was used to evaluate the method error. *P* value < 0.05 was set as statistically significant.

## Results

### Reliability of the measurements

The average intra-operator and inter-operator ICC (average ± SD, range) scored high: 0.987 ± 0.018, 0.937–0.995 and 0.968 ± 0.017, 0.931–0.984 respectively. According to Dahlberg’s formula, the random error for linear measurements was about 0.13 mm for the skeletal measurements, 0.26 mm for the dentoalveolar measurements, and 0.08 mm for the periodontal one.

### Baseline comparison

The demographic characteristics of subjects (age, sex, and cervical vertebra maturation stage) at baseline are summarized in Table 2. *T*-test and chi-square test demonstrated no significant differences between the Leaf Expander and RME groups for any of the measurements at the beginning of the treatment.

**Table 2:** Demography and clinical characteristics of the patients with the relative statistical analysis calculated by means of *t*-test for the age group comparison and chi-square test for differences in proportion

Sample characteristics	Total (*N* = 47)	Leaf group (*N* = 23)			RME group (*N* = 24)	Significance (*p* value)
Mean age ± Standard deviation	8.1 ± 0.9	7.9 ± 0.7			8.2 ± 0.8	0.181
Sex						
Male	23	11			12	0.882
Female	24	12			12	
Stage of development						
CVMS I	31		15	16		0.619
CVMS II	16		9	7		

^*^Italicized values signify significant difference between groups (*p* value < 0.05)

Descriptive statistics and statistical pre-treatment comparison of the skeletal, dentoalveolar, and periodontal variables are reported in Table 2.

None of the variables showed any statistically significant difference between the two groups and demonstrated similar baseline characteristics of sex, age, and the presence of clinical maxillary deficiency Tables 2, 3).

**Table 3:** Descriptive statistics with mean value ± standard deviation (SD) and independent sample* t* test for pre-treatment comparisons of Ni–Ti leaf springs expander (Leaf Expander) groups and rapid maxillary expansion (RME)

	Leaf expander group		RME group		
Variables	Mean	SD	Mean	SD	*p* value
Skeletal					
PNW (mm)	28.03	2.53	26.97	2.11	0.13
PABW (mm)	57.72	3.05	56.19	3.61	0.18
MW (mm)	60.63	3.39	59.21	3.05	0.15
MMW (mm)	25.11	3.15	24.39	2.69	0.40
PD (mm)	12.52	1.86	13.7	2.34	0.06
Dentoalveolar					
PAPW (mm)	53.62	4.10	52.96	3.89	0.57
UIMW (mm)	44.49	3.35	43.87	3.72	0.58
USIMW (mm)	45.44	2.91	45.10	2.38	0.66
LIMW (mm)	43.05	2.54	42.81	2.91	0.76
UICW (mm)	29.22	2.08	28.48	2.75	0.30
LICW (mm)	24.85	1.83	23.97	1.39	0.07
UMAA—Right (°)	77.31	6.32	78.29	7.21	0.62
UMAA—Left (°)	76.21	6.20	75.53	7.13	0.73
UAP (mm)	95.71	6.23	93.94	5.45	0.31
LAP (mm)	93.10	5.18	92.34	5.71	0.64
UCI (°)*	109.28	8.23	111.01	10.41	0.53
Periodontal (Buccal alveolar bone)					
BABT-Right (mm)	2.41	0.39	2.34	0.51	0.08
BABT-Left (mm)	2.24	0.45	1.99	0.56	0.32
BABH-Right (mm)	7.29	0.71	6.95	0.57	0.08
BABH-Left (mm)	7.66	0.65	7.43	0.69	0.25

^*^Italicized values signify significant difference between groups (*p* value < 0.05)

^#^Mean value between right and left side

### Skeletal changes

The paired sample *t*-test showed a statistically significant increase (*p* < 0.001) in the Leaf Expander group between T1 and T2 for the maxillary width (MW), whereas patients treated with RME protocols demonstrated a significant increase (*p* < 0.05) for all the skeletal variables considered (PNW, PABW, MW, and MMW) with the exception of the palatal depth (PD) (Table 4).

**Table 4:** Descriptive statistics with mean value ± standard deviation (SD) and Student’ paired t-test. Comparison between pre-treatment (T1) and post-treatment (T2) changes after Ni–Ti leaf springs expander (leaf) and rapid maxillary expansion (RME)

	Leaf group					RME group				
	T1		T2		Significance	T1		T2		Significance
Variables	Mean	SD	Mean	SD	*p* value	*Mean*	*SD*	*Mean*	*SD*	*p* value
Skeletal										
PNW (mm)	28.03	2.51	29.25	2.12	0.11	26.97	2.11	29.03	1.63	< 0.01*
PABW (mm)	57.72	3.05	59.55	3.23	0.08	56.19	3.61	58.79	3.8	0.03*
MW (mm)	60.63	3.38	62.82	3.12	< 0.001*	59.21	3.05	62.15	4.08	< 0.001*
MMW (mm)	25.11	3.15	26.84	2.71	0.06	24.38	2.69	27.19	2.45	< 0.01*
PD (mm)	12.52	1.86	12.0	2.22	0.43	13.72	2.34	12.63	2.74	0.17
Dentoalveolar										
PAPW (mm)	53.62	4.10	56.99	4.24	< 0.001*	52.96	3.89	57.30	4.11	< 0.001*
UIMW (mm)	44.45	3.35	47.88	3.75	< 0.01*	43.87	3.72	47.98	4.27	< 0.01*
USIMW (mm)	45.44	2.91	46.83	3.11	0.14	45.10	2.38	47.41	2.85	< 0.05*
LIMW (mm)	43.05	2.54	44.74	2.68	0.03*	42.81	2.91	44.95	3.44	0.02 *
UICW (mm)	29.22	2.08	34.62	2.33	< 0.01*	28.48	2.75	34.29	2.93	< 0.01*
LICW (mm)	24.85	1.83	25.01	1.73	0.67	23.97	1.39	24.22	2.67	0.77
UMAA—Right (°)	77.31	6.32	76.43	5.92	0.25	78.29	7.21	76.8	6.8	0.35
UMAA—Left (°)	76.21	6.20	72.05	7.11	0.32	75.53	7.13	70.6	8.9	0.47
UAP (mm)	95.71	6.23	100.91	5.42	0.03*	93.94	5.45	100.43	6.33	0.02*
LAP (mm)	93.10	5.18	93.44	5.48	0.53	92.34	5.71	92.99	4.93	0.33
UCI (°) ^#^	109.28	8.23	106.77	5.82	0.03*	111.01	10.41	108.45	8.37	0.04*
Periodontal (buccal alveolar bone)										
BABT-Right (mm)	2.41	0.39	2.18	0.35	0.103	2.35	0.54	2.19	0.39	0.27
BABT-Left (mm)	2.23	0.47	2.09	0.39	0.078	1.97	0.58	1.84	0.50	0.47
BABH-Right (mm)	7.29	0.71	7.79	0.74	0.179	6.99	0.58	7.34	0.61	0.08
BABH-Left (mm)	7.63	0.68	8.05	0.93	0.115	7.45	0.71	7.87	0.95	0.20

^*^Bold values signify significant difference between T1–T2 (*p* value < 0.05)

^#^Mean value between right and left side

Independent sample *t*-tests used for the group comparisons highlighted how patients treated with RME exhibited statistically larger width increases than those in the Leaf Expander group of 0.92 mm (*p* = 0.032) in terms of the posterior nasal width (PNW) and the maxillary mid-alveolar width (MMW) 0.91 mm (*p* = 0.022) (Table 5).

**Table 5:** Descriptive statistics with mean value ± Standard deviation (SD) and independent student’ *t*-test. Comparison of the changes occurred after Ni–Ti leaf springs expander (leaf) and rapid maxillary expansion (RME)

	Leaf group		RME group			
	Δ (T2–T1)		Δ (T2–T1)			Significance
Variables	Mean	SD	*Mean*	*SD*		*p* value
Skeletal						
PNW (mm)	1.22	1.19	2.14	1.41		0.03*
PABW (mm)	1.83	2.55	2.60	2.14		0.25
MW (mm)	2.17	1.44	2.99	3.35		0.28
MMW (mm)	1.73	1.44	2.81	1.72		0.02*
PD (mm)	−0.52	1.88	−1.09	2.1		0.41
Dentoalveolar						
PAPW (mm)	3.36	1.88	4.07		1.27	0.14
UIMW (mm)	3.43	2.17	4.11		2.43	0.84
USIMW (mm)	1.41	1.16	2.30		1.68	0.37
LIMW (mm)	1.69	1.07	2.14		0.87	0.62
UICW (mm)	5.40	1.34	5.81		1.60	0.13
LICW (mm)	0.16	0.72	0.25		0.97	0.72
UMAA—Right (°)	−1.50	5.6	−4.90		8.4	0.11
UMAA—Left (°)	−2.21	5.30	−1.72		5.50	0.49
UAP (mm)	5.21	3.72	6.49		2.60	0.21
LAP (mm)	0.34	2.64	0.65		2.20	0.34
UCI (°) ^#^	−2.62	4.93	−2.64		10.34	0.11
Periodontal (buccal alveolar bone)						
BABT-Right (mm)	−0.23	0.19	−0.16		0.16	0.25
BABT-Left (mm)	−0.14	0.20	−0.13		0.12	0.41
BABH-Right (mm)	0.50	0.32	0.35		0.28	0.22
BABH-Left (mm)	0.42	0.21	0.42		0.41	0.67

^*^Italicized values signify significant difference between T1 and T2 (*p* value < 0.05)

^Δ^Difference between T1 and T2

^#^Mean value between right and left side

### Dentoalveolar changes

A statistically significant increase (*p* < 0.05) was found in the Leaf Expander group for the following dentoalveolar measurements: PAPW, UIMW, LIMW, UICW, UAP, and UCI as reported in Table 4.

Subjects that underwent RME therapy showed a statistically significant increase (*p* < 0.05) for: PAPW, UIMW, USIMW, LIMW, UICW, UAP, and UCI.

Treatment comparisons between the Leaf Expander and RME groups are presented in Table 5. No statistically significant difference was noticed when comparing all the dentoalveolar parameters.

### Periodontal bone changes

Descriptive statistics and statistical comparison regarding buccal bone plate changes in both maxillary expansion protocols are summarized in Tables 4 and 5.

Neither the buccal alveolar bone thickness (BABT) nor the buccal alveolar bone height (BABH) showed a statistically significant difference (*p* > 0.05) after treatment with Leaf expander and RME and no statistically significant difference were found between the two groups.

## Discussion

To date, no studies in literature have assessed by means of CBCTs the skeletal, dentoalveolar, and periodontal effects of the Leaf expander. Furthermore, only a few published articles have begun to describe the three-dimensional quantitative changes (at the skeletal, dentoalveolar, and periodontal level) following rapid and slow maxillary expansion protocols with mixed results being highlighted [8, 37, 38]. In the present study, the SME was obtain using the Leaf Expander as it provides calibrated and steady forces to perform maxillary expansion. The findings of the present CBCT-based evaluation corroborate the effectiveness of Leaf Expander and RME in patients during mixed dentition. The null hypothesis was rejected as a statistically significant difference was found for the skeletal variables. Concerning the latter, only PNW and MMW showed to be significantly greater in the RME group and these differences were lower than 1 mm.

It has been suggested that RME maximizes skeletal effects and minimizes dental ones [39]; however, many studies have demonstrated side effects associated with RME such as relapse of the expansion, tipping of the molar axes, reported pain, root resorption and, lastly, buccal tipping of the alveolar bone [40, 41]. The available evidence reports that both types of expanders (Leaf Expander and RME) provide effective maxillary expansion [18].

Concerning the slow maxillary expansion, some studies have shown that it produces less tissue resistance around the circummaxillary structures, thus allowing more adjustment to sutural separation, and improving bone formation in the intermaxillary sutures: this determines greater sutural stability, by reducing the post-expansion relapse of the RME [42–44].

Considering the skeletal changes occurred in the Leaf Expander group, a statistically significant difference was found between T1 and T2 for the maxillary width (MW) only. Conversely, patients treated with RME evinced a statistically significant increase for all the skeletal variables considered (PNW, PABW, MW, MMW) with the exception of the palatal depth (PD). Despite this, when compared, only the posterior nasal width (PNW) and the maxillary mid-alveolar width (MMAW) demonstrated to be significantly greater in the RME than with the Leaf Expander. Paoloni et al. [20] reported a significantly greater increase for maxillary width (MW) with the RME, which was not found in our study. Nonetheless, our findings are partially in agreement with those of Paoloni et al. [20] confirming a greater, although slight, skeletal expansion in favor of the RME therapy.

Lanteri et al. [18] reported no significant differences between Leaf Expander and RME for each of the skeletal and dento-alveolar parameters calculated on postero-anterior radiographs. The differences between this study and that done by Lanteri et al. [18] could be attributed to the limitations due to bidimensional radiographs and an extremely limited sample size (10 per group).

The results concerning dentoalveolar changes exhibited no statistically significant difference between Leaf Expander and RME for all of the measurements. The present findings corroborate the data previously reported in other systematic reviews where no significant difference comparing dentoalveolar transversal changes of the SME with the RME were reported [11].

Both of the appliances demonstrated a significant improvement for most of the dentoalveolar variables as reported in Table 4.

The Leaf Expander and RME showed a statistically significant increase at the level of the posterior alveolar process width (PAPW), upper intermolar width (UIMW), lower intermolar width (LIMW), and upper intercanine width (UICW). The results of the present study disagree with a randomized controlled trial performed on bidimensional radiographs and digital models by Paoloni et al. [20] where a significantly greater increase in the deciduous intercanine width (53–63) using RME was found. The present findings are also in contrast with those reported by Cossellu et al. [45] where a statistically significant difference in deciduous intercanine width (53–63) between Leaf Expander and RME were reported, finding significantly greater results in the Leaf Expander group, whereas the increase in maxillary intermolar width (16–26) was statistically significantly greater in the RME group. In this study no statistically significant difference was found between the two expansion protocols for the same variables. The different results of the ICW and IMW could be attributed to the different appliance design of the Leaf and RME expanders. In the study by Paoloni et al. [20]; in fact, the Leaf expander was not extended to the lingual face of the maxillary primary canines while the RME Expander used by Cossellu et al. [45] presented lingual wire extensions between the maxillary permanent first molars and the maxillary primary canines. When using the Leaf Expander the authors strictly recommend the utilization of the lingual wire extension to the maxillary primary canines to obtain a satisfactory increase in the inter-canine distance.

Only a few studies have evaluated the changes both on the maxillary and the mandibular arches after maxillary expansion [45–47]. In this research, the Leaf Expander and RME have demonstrated a statistically significant mandibular spontaneous response with an increase in the lower intermolar width (LIMW). The increase in the LIMW could be attributed to the augmented tongue pressure due to the presence of the appliance that influences a lower position of the tongue, a reduced lip, and cheek pressures and to the onset of new occlusal contacts (occlusion between the palatal cusp of upper first molars and the buccal cusp of the lower first permanent molars) [48]. Only a slight difference with no statistical significance was found at the level of the lower arch perimeter (LAP) that increased less than 1 mm in both groups. This data agrees with those previously published in the existing scientific literature [45–47].

The present study found also a significant spontaneous retraction of the upper permanent incisors with a decreased of the U1 ∧ SNP-SNA angles (UCI) with no differences comparing the two expansion modalities. These results agree with those previously published in the literature where a significant posterior movement of the upper incisors following Leaf Expander and conventional RME treatment were reported [27, 49].

A recent meta-analysis performed by Rutili et al. [50] reported that both RME and SME yield an efficient skeletal and dento-alveolar maxillary expansion. Moreover, the authors indicated a slight but more effective increase in the maxillary posterior skeletal width after RME while SME produced less molar tipping. The aforementioned findings are in agreement with those reported in the present research. It should be pointed out that in this study the SME was obtained with low compliance Ni–Ti leaf spring expanders that produced continuous and calibrated forces and not with the same screw expander that used a slower activation protocol as performed in the studies present in the systematic review.

It has been shown that both RME and SME cause orthopedic changes, dentoalveolar changes, and varying degrees of buccal bone loss [51, 52]. Moreover, recent researches and systematic reviews have confirmed potential periodontal and endodontic damage of RME when the first permanent molars are used as anchoring teeth [19, 53, 54]. Therefore, in the present study, the appliances were anchored to the deciduous teeth as suggested by some authors [31, 53, 54]. Despite this, the authors decided to investigate possible periodontal bone changes at the level of the first permanent molars after Leaf Expander and conventional RME. As the authors expected, no differences were found between the two timepoints after both therapies, and no statistically significant difference was found when the two groups were compared. This data confirmed the absence of significant loss of buccal bone thickness and height around the first maxillary permanent molars when palatal expansion is obtained using deciduous second molars as anchoring teeth using both Leaf Expander and RME.

Concerning the reason of the inclusion criteria to perform the second CBCT (T2) is that the last screw activation of the RME needs at least 6 months to steady the orthopedic effects. Moreover, according to the literature the average duration period of the active treatment and retention period lasts roughly 7/8 months and the mean treatment time using the Leaf Expander lasts 9 months [17]. The reason behind the age group selection is twofold; the first is that patients between the ages of 7 and 10 years old mainly have the second primary molars which offer an affective free anchorage, thus protecting the periodontal status of the first permanent molar; the second is that the prepubertal phase of development is the most suitable time to perform palatal expansion. In fact, as reported by Baccetti et al. [30], subjects treated during this stage of development (CVMS 1 and 2) demonstrate larger and more stable skeletal effects.

Limitations of the present research were the retrospective design of the study and the lack of long‐term follow‐up. Retrieving multiple scans of a patients over time allowed us to better understand the possible three-dimensional longitudinal changes over time such as eventual skeletal and dental relapse or alveolar bone damages. Further prospective CBCT studies, with relevant sample sizes and long-term examinations, are necessary to quantitatively evaluate the skeletal and dento-alveolar changes after Leaf Expander and RME treatment. However, the unjustified use of CBCT, due to the risks related to exposure to ionizing radiation, is strongly contraindicated, especially for pediatric examination, as reported by the DIMITRA guidelines [55] and the recommendations of the British Orthodontic Society and the American Association of Orthodontists [56, 57]. The continuous development of 3D radiation-free examinations such as magnetic resonance imaging (MRI) will hopefully remedy to this problem in the near future [22].

## Conclusions

Among the limitations of this study, the results of this research confirm the effectiveness of Leaf Expander and RME in treating maxillary deficiencies in mixed dentition patients. Concerning the skeletal parameters, only PNW and MMW showed to be significantly greater in the RME group. These differences could be considered irrelevant from the clinical point of view as they were lower than 1 mm. With regards to the considered dentoalveolar variables, no statistically significant differences between the Leaf Expander and the conventional RME were found. Moreover, both devices anchored to deciduous teeth did not impair the thickness and height of the buccal bone plate at the level of the first maxillary permanent molars. Therefore, the Leaf Expander appears to be a valid alternative to RME in the maxillary expansion therapy with respect to the evaluated variables.

## Data Availability

The data underlying this article will be shared on reasonable request to the corresponding author.
